# Missing and murdered indigenous women and girls in Canada: a new population affinity assessment technique to aid in identification using 3D technology

**DOI:** 10.1080/20961790.2021.2023417

**Published:** 2022-03-25

**Authors:** Elisabeth Cuerrier-Richer

**Affiliations:** Department of Anthropology, University of Toronto, Toronto, Ontario, Canada

**Keywords:** Forensic sciences, forensic anthropology, population affinity assessment, Canadian Indigenous, morphological analyses, cranial traits, 3D shape analysis

## Abstract

As of 2015, 204 cases of missing and murdered Indigenous women and girls (MMIWG) remained unsolved in Canada, making it a major concern for Canadian Indigenous communities, who are still pressing for the resolution of these cases. In forensic anthropology, the assessment of population affinity can be useful to help identify victims. Population affinity, previously referred to as ancestry, is evaluated based on morphological analyses, which examine the size and shape of skeletal features, and metric analyses, which utilise skeletal measurements. However, morphological analyses strongly depend on an anthropologist’s experience with human variation, which makes the analyses particularly challenging to reproduce and standardise. The purpose of this study is to improve the rigour of morphological analyses by using 3D technology to quantify relevant cranial nonmetric population affinity traits. As there is currently little morphological data available for the Canadian Indigenous population, this research aims to develop a new technique that could aid in the identification of MMIWG. The study comprised a total of 87 adult female crania, including 24 of Canadian Inuit origin, 50 of European descent and 13 of African descent. The samples were imaged using photogrammetry, then analysed using a 3D shape analysis in 3DS Max. Results show that this method is satisfactory in correctly evaluating population affinity with an accuracy of 87.36% (jackknifed: 80.46%) and an average repeatability of 97%. Unfortunately, the small Canadian Indigenous sample size impacted the applicability of the results and further research will be required before the technique can be used to aid in the identification of MMIWG in Canada.

## Key points

- MMIWG are over-represented as victims of violence and homicide in Canada.
- Population affinity provides biogeographic data to help search national databases.
- Cranial morphological variation can be quantified in 3D accurately and precisely.

## Introduction

According to the Royal Canadian Mounted Police (RCMP), women of Canadian Indigenous descent, previously referred to as Aboriginal and comprised of individuals identifying as First Nations (North American Indian), Inuit and Métis [1], are over-represented as victims of violence and homicide [2, 3]. In 2013, an extensive study into the RCMP’s reported incidents of missing and murdered Indigenous women and girls (MMIWG) across all police jurisdictions in Canada was mandated by the Commissioner of the RCMP. The results of this study were published in a national operational overview in 2014 [2] and updated in a follow-up report in 2015 [3]. The fact that Indigenous women and girls have higher victimisation rates than other women in Canada was supported by numerous findings. In 2015, Indigenous women and girls accounted for 24% of female homicide victims in Canada, an increase from only 9% in 1980 [4]. From 1980 to 2012, a significant number of Indigenous female homicides were observed in the provinces of Alberta (28% of all female homicides), Manitoba (49%) and Saskatchewan (55%). The highest rates were concentrated in Northern Canada, in Yukon (56%), the Northwest Territories (92%) and Nunavut (100%) [2]. With regards to missing Indigenous women and girls, 174 Aboriginal females were reported on the Canadian Police Information Centre (CPIC) as missing for more than 30 days as of April 2015. Of this number, 111 cases were categorised as “unknown” or suspected foul play [3]. However, the data do not include missing females who may not have been identified as Aboriginal in case reports or disappearances who were not reported, so it is likely the true number of missing Indigenous women and girls is higher [2]. Over the last four decades, every province and territory has been touched by this issue, leading to a national crisis that has affected hundreds of families. As of 2015, 204 cases of MMIWG remained unsolved, making it a major concern for Canadian Indigenous communities, who are still pressing for the resolution of these cases [4].

While they provide important statistical data into the MMIWG issue, the RCMP’s reports [2, 3] do not address cases that may have used the skeletal analysis of human remains to aid in identification. When human remains are found, they may be in an advanced state of decomposition or skeletonised. Under such circumstances, forensic anthropologists can help identify victims by establishing a biological profile of the skeleton. This profile includes the analysis of sex, population affinity, age and stature from skeletal remains, which allows investigators to narrow down the list of potential matches in missing persons files [5]. In the current context, “population affinity” refers to an individual’s biogeographic region of origin. Population affinity assessments can therefore help direct the search in national missing persons databases by providing the appropriate biogeographic information needed to further reduce the number of possible matches [5]. In the case of MMIWG, identifying a Canadian Indigenous origin early in the case can accelerate the search process and shift the focus quickly to the appropriate authorities. Unfortunately, skeletal data from individuals of Canadian Indigenous biogeographic origin are currently limited, an issue that affects a forensic anthropologist’s ability to provide more specific biogeographic information in ongoing cases.

Population affinity assessments from the skeleton are possible, yet complex because human variation exists along a geographic continuum and is shaped by evolutionary processes, such as natural selection, mutation, gene flow and genetic drift [5]. Population affinity, previously referred to as ancestry, is evaluated using a two-part approach: morphological analyses, which examine the size and shape of skeletal features, and metric analyses, which employ skeletal measurements [6]. Morphological analyses, also known as nonmetric or anthroposcopic analyses, evaluate skeletal traits according to the type of analyses performed: binary, where the trait is present or absent, or morphoscopic, where the trait can have different states depending on its shape or degree of expression [7]. In the past, forensic anthropologists have relied on a list of traits that they believed could discriminate between populations, as various character states for each trait were assigned to different groups [8]. For example, individuals of European descent were described by Rhine [9] as having sloped orbits, a narrow nasal aperture and a parabolic dental arcade. Those of African descent exhibited high frequencies of rectangular orbits, a wide nasal aperture and a hyperbolic dental arcade [9]. Finally, people of Asian/Indigenous descent were described as having rounded orbits, an intermediate-sized nasal aperture and an elliptic dental arcade [9].

The relationship between biological and social aspects of population affinity and ethnicity is complex and poses a challenge for forensic anthropologists attempting to provide a biological description of human skeletal remains for the purpose of identification in medico-legal contexts. Of concern are situations where individuals may identify, or be identified by others, with a different social group than their biological origin [7]. This disparity becomes problematic when a missing individual’s population affinity, as reported to the police, does not correspond to the assessment made by the forensic anthropologist. To be understood by law enforcement personnel and the general public for investigative purposes, a forensic anthropologist’s description of population affinity in the biological profile must include terms that closely correspond to socially familiar terminology, which are often associated with “race” [7, 10]. In anthropology, the term “race” is defined as a sociocultural construct without a biological factor that represents an individual’s self-identification and group membership [11–13]. In contrast, the definition employed by the general public combines biological components, such as anatomical features that individuals share through common heredity [14], as well as components of ethnicity, which are generally reflected through nationality, shared languages, religion, customs, beliefs, etc. [11]. Although reporting population affinity in socially understandable terms can be beneficial to the investigation, anthropologists have been strongly criticised over the last few decades for encouraging the categorisation of individuals through their population affinity assessments [e.g. 15]. Forensic and biological anthropologists alike have therefore progressively clarified the distinctions between the terms population affinity, ethnicity and “race”, and have distanced themselves from the morphological “trait list” [11–14].

Although some of its traits are still used today by forensic anthropologists to help orient the assessment of population affinity, the “trait list” is difficult to apply, and thus, highly problematic. This approach lacks statistical rigour, as it is not possible to calculate an error rate nor quantify the assessments [10]. In this situation, one cannot subsequently determine the validity or reliability of the results obtained. Furthermore, the morphological approach depends on an anthropologist’s experience with human variation, which makes the resulting analyses highly subjective and consequently, very challenging to reproduce and standardise [6]. A lack of standardisation in forensic science is a critical issue that was addressed in the Daubert court ruling of 1993 [16] and the U.S. National Academy of Sciences report of 2009 [17]. Multiple authors [6, 8, 10, 18] have heavily criticised the use of the “trait list” and have searched for new techniques that would offer a more standardised and objective evaluation of population affinity. However, such methods rely on traditional morphological and metric assessments on the original bones. Forensic anthropologists may encounter situations where the skeletal material is very fragile, such as in fire cases [19], or no longer accessible, e.g. if the deceased has been returned to the family. Under such circumstances, traditional assessments may be difficult, if not impossible, to perform. As medical and forensic agencies transition towards a more consistent use of three-dimensional (3D) technology for autopsies [20] and crime scene reconstructions [21], assessment techniques employing 3D models are also becoming more relevant for documentation and analysis.

The purpose of this study is to improve the rigour of morphological analyses by using 3D technology to quantify relevant cranial nonmetric population affinity traits on individuals of Canadian Indigenous, European and African biogeographical origin. More specifically, circumference/perimeter, surface area of the defined perimeter and proportion ratios are used to characterise and quantify the 3D outline of the orbits, the nasal aperture and the palate, in lieu of the current subjective criteria (e.g. “round” *versus* “square” orbits). As there is currently little morphological data available for the Canadian Indigenous population, this research aims to develop a new technique that could aid in the identification of MMIWG. Individuals from two commonly studied populations (European and African biogeographical origin) are also included to establish a complementary method of analysis for cases in which 3D imaging is a possible, if not preferred, method of documentation.

## Materials and methods

### Samples

The study comprised a total of 87 adult female crania, including 24 of Canadian Inuit origin, 50 of European descent and 13 of African descent. Sex, population affinity and age for all individuals in this study are available in Table 1.

**Table 1.** Sample distribution (*n*, %) by population and average age.

Population	Average age (years)	No. (%) of individuals
African	60	13 (14.94)
European	66	50 (57.47)
Canadian Inuit	Adult	24 (27.59)
**Total**	63*	**87 (100.00)**

*Not an accurate representation due to the lack of accurate age for Canadian Inuit.

Biological females were the focus of this research in order to develop a technique for the identification of MMIWG, as well as to control for sex differences. The Canadian Inuit sample was derived from the Canadian Indigenous collection curated at the Canadian Museum of History (CMH) in Gatineau, Québec (Canada). Individuals originated from four archaeological sites in the Northwest Territories dated from the historic period (J. Young, personal communication, May 23, 2018); specific site information is indicated in Table 2. The individuals’ sex had been assessed previously by curators at the CMH and was confirmed by the author using traits by Williams and Rogers [22] prior to digital imaging. Unfortunately, the individuals’ age was unknown and had not been assessed previously by CMH curators, leading to age assignments being separated as adult or juvenile only in the museum’s database (J. Young, personal communication, May 23, 2018). The distinction between adults and juveniles was confirmed by the author prior to imaging. Although the issue of MMIWG also includes girls, only adult individuals were included to remain consistent with the other samples available and to avoid variation brought about by immature cranial morphology. The current study followed all protocols and procedures set out by the CMH regarding Canadian Indigenous’ permissions for research. Data collection at the CMH took place in October 2018.

**Table 2.** Canadian Inuit sample distribution (*n*, %) by archaeological site (*N* = 24).

Archaeological site	Borden No.	Collection year	No. (%) of individuals
Fort Simpson	JlRi-2	1966	1 (4.17)
Kiklewait	NjTr-1	1933	1 (4.17)
Richards Island	Unknown	1934	2 (8.33)
Kittigazuit	NiTr-2	1935, 1969	20 (83.33)*

*Specifically 9 individuals in 1935 and 11 in 1969.

The individuals of European and African descent were housed in the William M. Bass Donated Skeletal Collection curated at the University of Tennessee’s Forensic Anthropology Centre (FAC) in Knoxville, Tennessee, USA. The collection houses individuals that represent the USA’s modern population, many of them originating from Tennessee and Southeastern US. Sex, population affinity, age, cause of death and body mass index (BMI) are known for most of the individuals in the collection [23]. Data collection at the FAC took place in August 2018.

### Digital imaging

The individuals were digitally imaged using photogrammetry techniques. Photogrammetry, defined as the measurement from photos, uses 2D photos of the subject taken from multiple angles and determines the subject’s 3D form through triangulation of common points found in two or more images, through the mathematical calculation of intersecting points [24]. The entire process of completing a 3D reconstruction with photogrammetry has been described in detail elsewhere [24–28], so the reader is referred to these authors for more information. Photographs were taken with a Nikon D7200 camera at a resolution of 24 megapixels. Additional photography equipment included an 18-140 mm Nikkor lens, a Godox TT350 external flash unit and a Benro TMA28A MACH3 Series 2 tripod with an HD2A head. Prior to imaging, the exposure of the camera was adjusted to provide images that were consistently balanced in terms of light [29].

**Figure 1.**
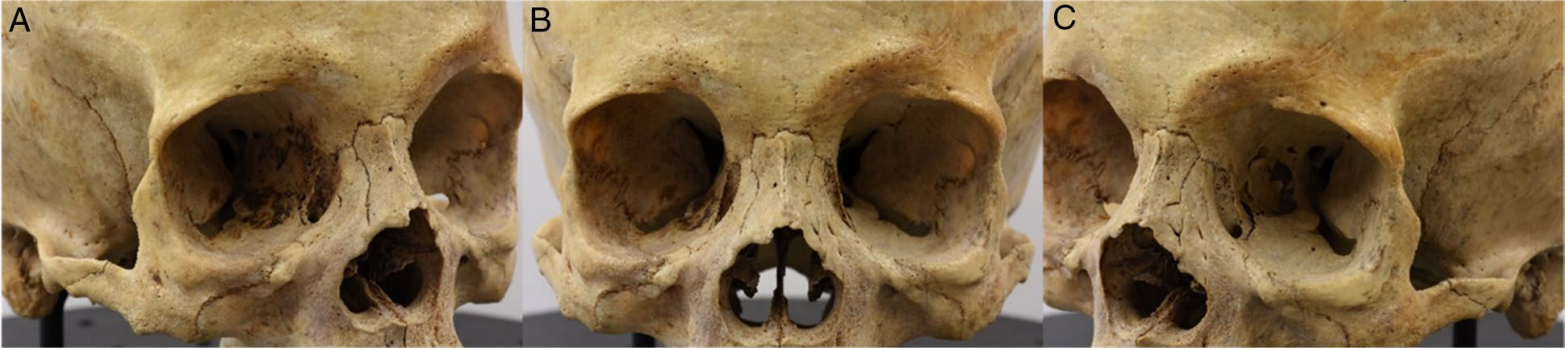
First (A), middle (B) and last (C) photos of the first set.

**Figure 2.**
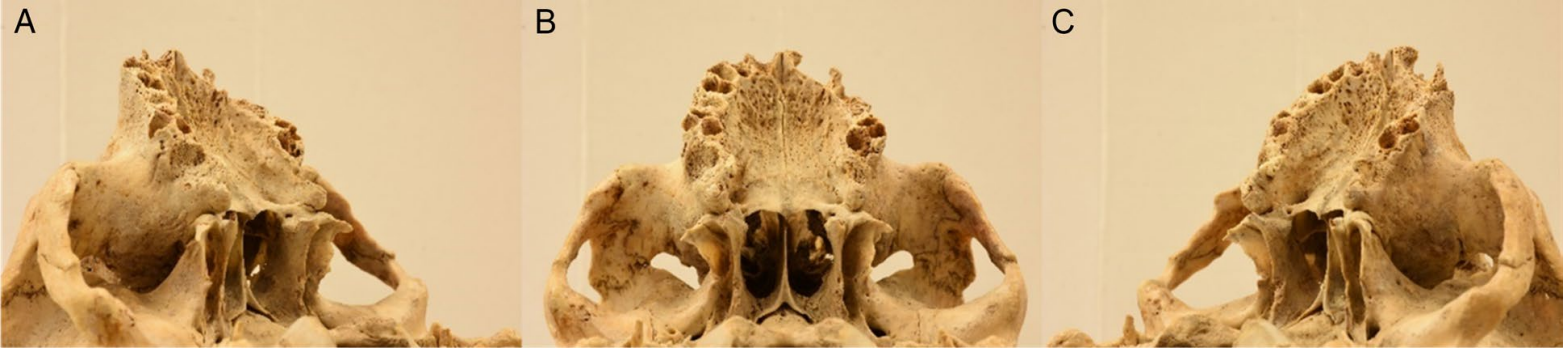
First (A), middle (B) and last (C) photos of the second set.

**Figure 3.**
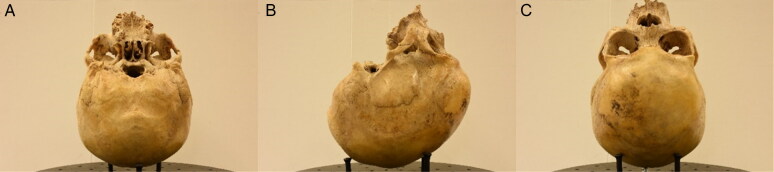
First (posterior view) (A), left lateral (B) and middle (anterior view) (C) photos of the third set.

**Figure 4.**
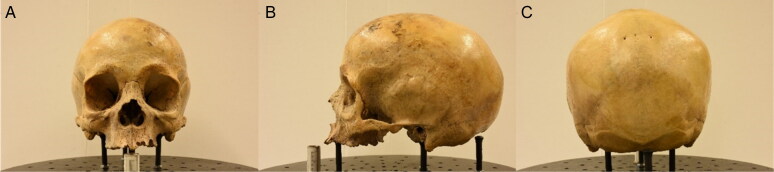
First (anterior view) (A), left lateral (B) and middle (posterior view) (C) photos of the fourth set.

**Figure 5.**
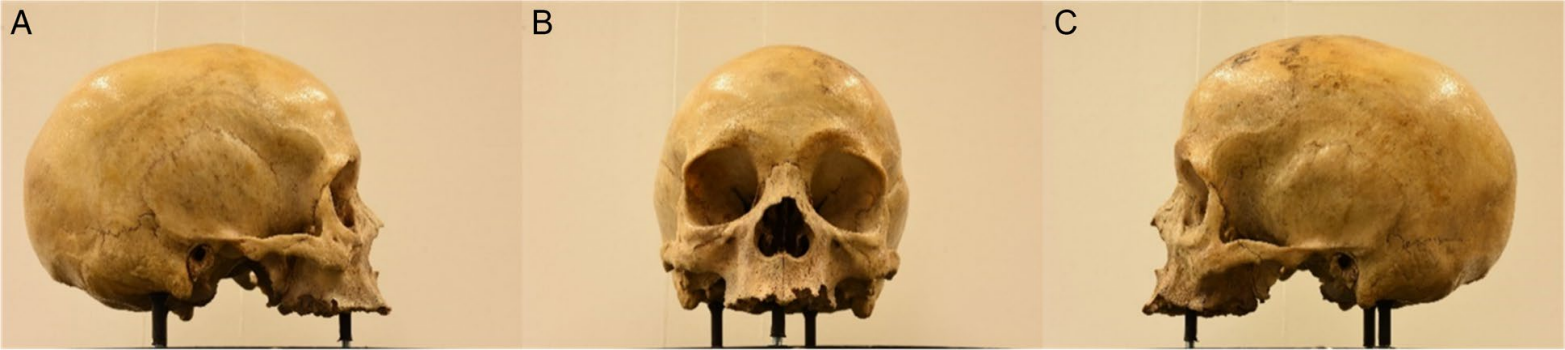
First (A), middle (B) and last (C) photos of the fifth set.

**Figure 6.**
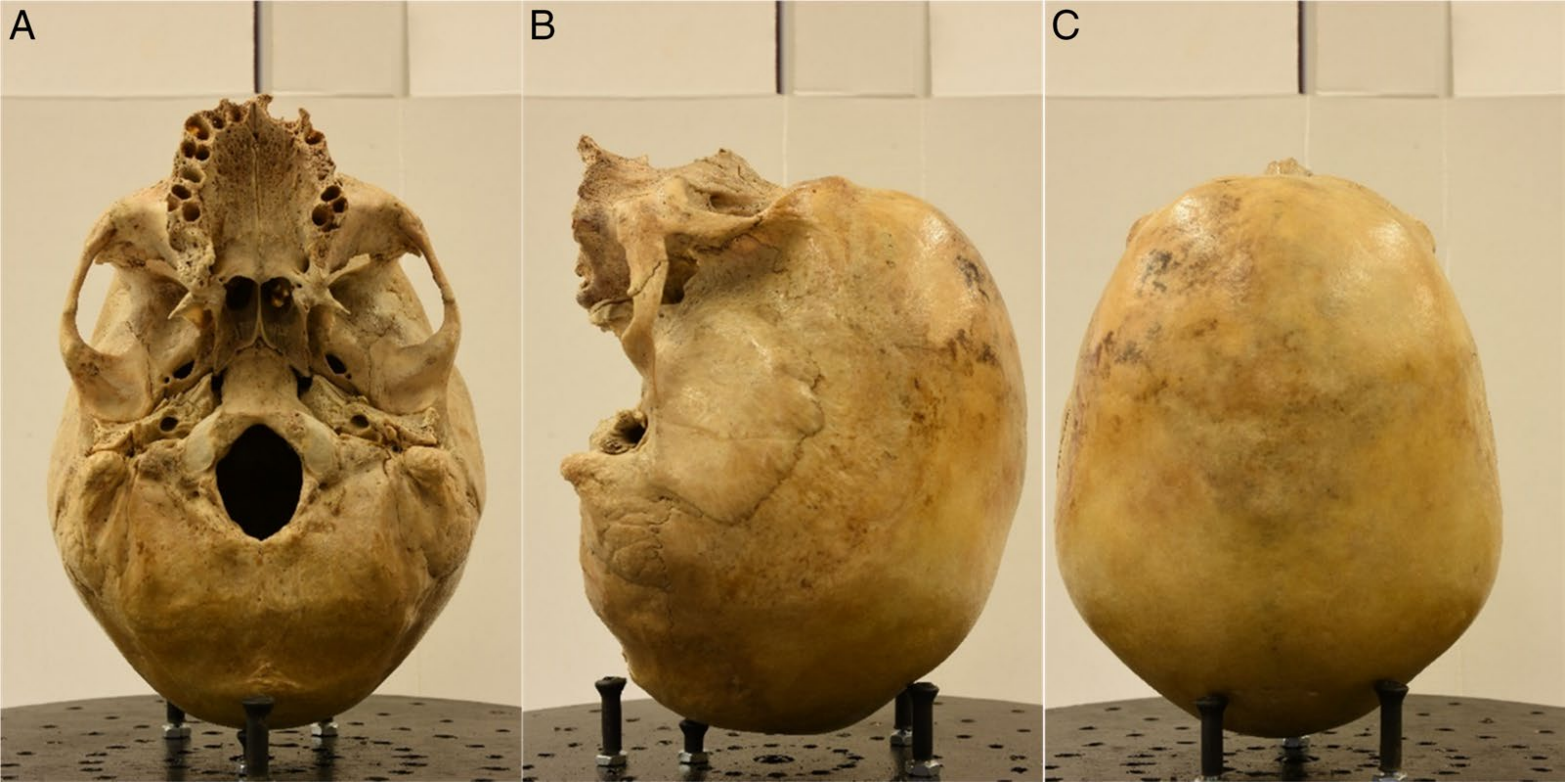
First (inferior view) (A), left lateral (B) and middle (superior view) (C) photos of the sixth set.

The workspace was standardised by having every cranium placed on a customised turntable approximately 30 cm from the camera. Each individual was imaged using the same procedure, comprised of six sets of photos, to ensure an adequate overlap of image content between photographs that would allow proper 3D reconstruction in the photogrammetric software afterwards (Figures 1–6). Depending on the image content desired, full 360° turns of the turntable were not always necessary and in certain sets, only a few sequential photos were taken (Table 3). The entire process took between 30 and 45 min and produced a total of 100 photos per individual.

**Table 3.** Photography procedure by set, including skull position, focal length, camera angle, view, number of photos and target views of the skull.

Set No.	Skull position (relative to turntable)	Focal length (mm)	Camera angle (°)	View (°)	No. of photos	Target views of skull
1	Horizontal (in Frankfort plane)	140	+10	60	5	Inferior border of orbits and anterior opening of nasal cavity
2	Inverted, facial skeleton at 45°∠	140	0	90	7	Interior of nasal cavity’s posterior opening
3	Inverted, facial skeleton at 45°∠	35	0	360	25	Palate, inferior borders of orbits, anterior opening of nasal cavity in superior view and posterior opening of nasal cavity in inferior view
4*	Horizontal	35	0	360	25	Full anterior, lateral & posterior views of skull
5	Horizontal	35	−10	180	13	Superior borders of orbits and anterior opening of nasal cavity in inferior view
6	Vertical (perpendicular to Frankfort)	140	90	360	25	Full superior and inferior views of skull

* A 20-mm scale was included to allow for calibration to a real-world scale in the photogrammetry software.

### Photogrammetry modelling

Once digital imaging was completed, the photographs were imported in JPEG format to the 3DF Zephyr Aerial 4.0 photogrammetric software (3Dflow, Verona, Italy), which aligned and merged the photos to create scaled 3D models with colour and texture. The 3D computations of this study were completed with an MSI GS63 7RD-072CA Stealth computer (Micro-Star International Co., Ltd., Taiwan, China), with specifications including a 2.80 GHz Intel Core i7-7700HQ processor, 16 GB of DDR4 RAM system memory, hybrid storage and a NVIDIA GeForce GTX1050 graphics card.

A 3D reconstruction using photogrammetry requires four phases, in addition to scaling (see [24–28] for more information). In this study, the entire 3D reconstruction process took on average 127 min per individual (Table 4). Scaling accuracy had an average of 0.0514 pixel for the European and African samples combined, and an average of 0.0416 pixel for the Canadian Inuit sample.

**Table 4.** 3D reconstruction times and resolutions per phase.

Phase	Average time* (min)	Resolution
Sparse reconstruction	23	63 000 sparse cloud points
Dense point cloud generation	58	2.9 million points
Mesh extraction	15	900 000 points / 1.8 million triangles (faces)
Textured mesh generation	31	900 000 points / 1.8 million triangles (faces)
**Total**	**127**	

* Per individual.

### 3D shape analysis

Once the 3D models were created in 3DF Zephyr and exported, they were imported to the Autodesk 3DS Max 2018 software (New York, NY, USA) for the shape analysis of the orbits, the nasal aperture, and the palate. Prior to working with the models, the 3D workspace was standardised by having the object’s pivot point centered to the 3D model, the model placed at the origin (0, 0, 0) and orienting the model so that the facial skeleton faced the screen in the Frankfort horizontal plane.

This research analysed the orbit, nasal aperture, and palate, which were chosen for specific reasons. The orbits and the palate are at an impasse, as conflicting results have been found in support of their use in population affinity assessment [6, 30]. As for the nasal cavity, its shape has been found to vary between populations of different climates [31–33], but a limited number of North American populations were considered in past studies and therefore remain to be assessed with the current research.

The traits were analysed to obtain circumference/perimeter, surface area of the defined perimeter and proportion ratios as 3D measurements for each trait. The palate was also subjected to a supplementary depth measurement, leading to a total of thirteen 3D measurements performed on all individuals. The circumference/perimeter and surface area measurements were mutually dependent and had to be done successively, while the proportion ratios and depth measurements did not have this requirement and can be done in a different order from the one presented here. To ensure that this approach could be undertaken by as many anthropologists as possible, basic tools from the 3DS Max software were used to acquire the 3D measurements.

#### Circumference/perimeter measurements

The “Line” tool was employed to trace the outline of the traits’ 3D shape. Once the line was closed (i.e. completely wrapped around the shape), the software provided a measurement representing the length of the line, which could then be translated to circumference values for the orbit, and perimeter values for the nasal aperture and palate.

**Figure 7.**
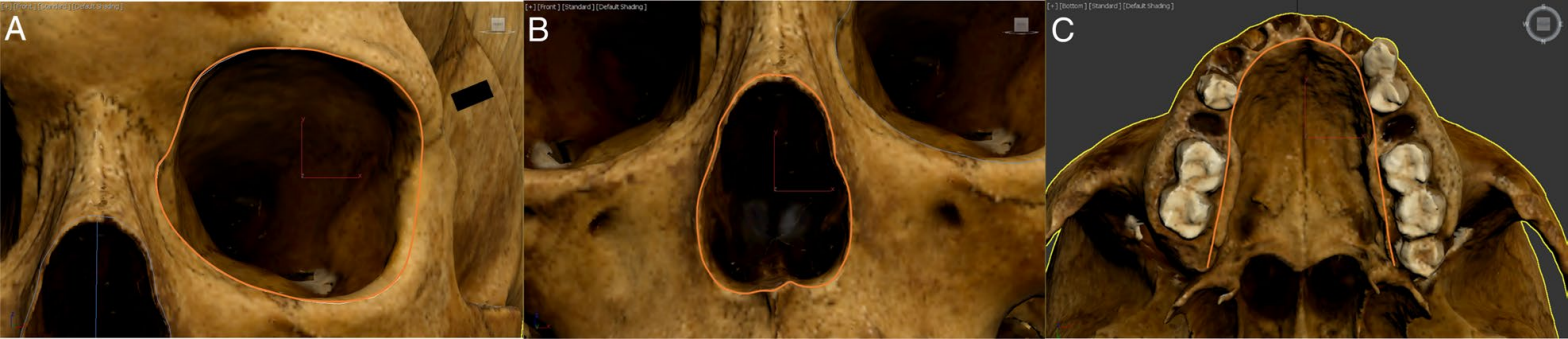
Examples of (A) left orbit’s circumference, (B) nasal aperture’s perimeter, and (C) palate’s perimeter measurement.

For the orbit, all measurements were performed on the left side for consistency; if the left orbit was damaged, measurements were performed on the right side. Tracing started at the frontomalare orbitale landmark, where the frontozygomatic suture crosses the inner orbital rim [34]. It progressed medially along the superior orbital rim towards the dacryon landmark, where the maxillolacrimale suture meets the frontal bone [34], before returning to frontomalare orbitale along the inferior orbital rim (e.g. orange outline in Figure 7A). An average of 35 vertices was used to trace the orbit for all samples in the study.

For the nasal aperture, tracing began at the most projecting point of the anterior nasal spine. It continued superiorly along the border of the nasal aperture towards the rhinion landmark, the midline point at the inferior free end of the internasal suture [34], before returning inferiorly along the border to the anterior nasal spine (Figure 7B). An average of 46 vertices was used to trace the nasal aperture.

**Figure 8.**
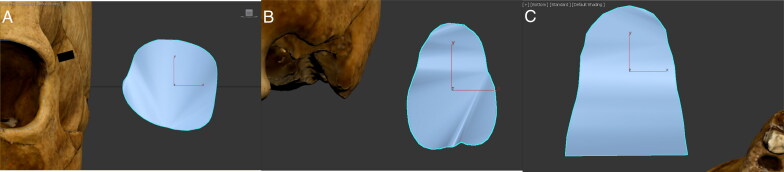
Examples of surface area measurement of (A) left orbit, (B) nasal aperture, and (C) palate.

For the palate, tracing commenced at the most posterior point of the alveolar process behind the M^3^ on the inner surface of the alveolar margin. It followed the inner alveolar margin, at the joint between the tooth root and the bone, until the most posterior point on the other side was reached (Figure 7C). An average of 25 vertices was used to trace the palate.

**Figure 9.**
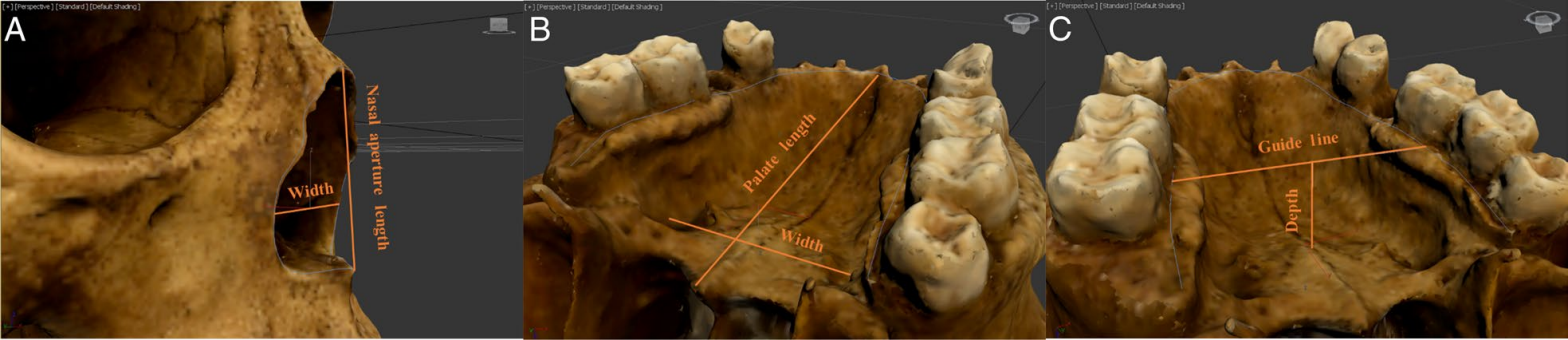
Examples of (A) nasal aperture’s length and width, (B) palate’s length and width, and (C) palate’s depth measurement.

#### Surface area measurements

The newly traced shapes were then converted to polygons using cloning and conversion features, which provided a surface area value for the traced shapes (Figure 8). These measurements specifically can inform on the subjective criteria currently used (e.g. “round” versus “square” orbits) and help establish how to quantify the observed human variation (e.g. what value can be associated with “round” orbits and what value can be associated with “square” ones).

**Figure 10.**
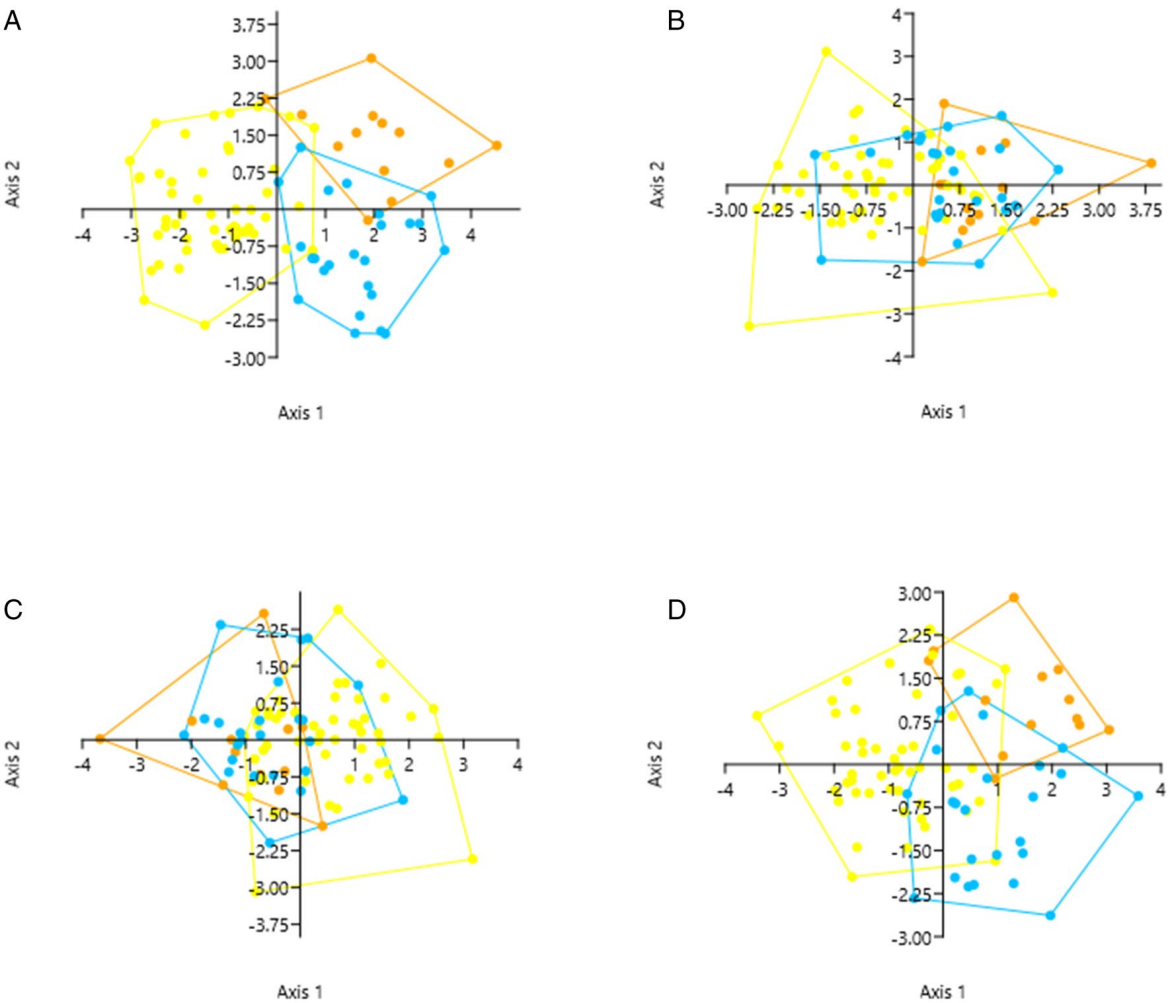
Discriminant function plots for 3D measurements combined (A), circumference/perimeter (B), surface area (C), and proportion ratios (D) (yellow: European; orange: African; blue: Canadian Inuit).

#### Proportion ratios measurements

The “Line” tool was also employed to measure linear distances in 3D space. The linear distances of length and width for the proportion ratios were taken between two points and the end of the measurement was accomplished by clicking the right mouse button. No proportion ratio was calculated for the orbit, as the models’ resolution made it difficult to consistently identify the appropriate landmarks from which to take measurements in all of the 3D models.

For the nasal aperture, the length was measured from the rhinion landmark to the most projecting point of the anterior nasal spine. These measurement points were chosen because they were consistent landmarks in all models. The width was measured as the nasal breadth (i.e. the distance between the most lateral points on the anterior nasal aperture’s margins) [34] (Figure 9A). The ratio was calculated as the length divided by the width.

For the palate, the length was measured from the point closest to the incisors, following the groove of the incisive foramen, to the alveolon landmark, which is the point on the intermaxillary suture where a line drawn between the posterior ends of the alveolar ridges crosses the line [34]. The width was measured along the transverse palatine suture, at the widest points of the alveolar margins when closest to the palate floor (Figure 9B). The ratio was calculated as the length divided by the width.

#### Depth measurement

Finally, the palate depth was measured at the intersection of the median palatine suture and the transverse palatine suture, perpendicular to a guiding line drawn across the alveolar margins, as close as possible to the joint between the tooth root and the bone (Figure 9C).

### Statistical analysis

Measurements were repeated on a subset including all populations and representing approximately 16% of the original sample to test for intra-observer error. A second repeat of the measurements was performed 2 weeks after the end of the first repeat. Intra-observer error was tested statistically using the repeatability equation described in Harper [35].

After testing for intra-observer error, all samples were subjected to a test of multivariate normality and Box’s M test to ensure that assumptions of multivariate normality and equality of covariance matrices were met [36]. As the multivariate normality and Box’s M test results were statistically significant (i.e. non-normal and non-equivalent), multivariate assumptions were not met. A non-parametric multivariate analysis of variance (PERMANOVA) was therefore used to determine if populations can be distinguished statistically at the significance level of 0.05 [36]. The PERMANOVA was followed by a discriminant function analysis (DFA) to evaluate where the variation lies within and between populations (i.e. how the groups differ) [37].

The analyses were first performed on all 3D measurements combined. However, as not all measurements were assessing the same shape components, the analyses were subsequently performed by measurement category, meaning that tests were also run individually for circumference/perimeter, surface area of the defined perimeter and proportion ratios.

Since a significant number of individuals were impacted by severe resorption of the dental arcade, the palate’s perimeter, surface area and depth measurements were inaccurate and had to be discarded from statistical analyses. The proportion ratios were not affected by the resorption and were therefore included as part of the study’s results.

The data entry was performed in Excel 2017, (Microsoft Corporation, Redmond, WA, USA) while all the multivariate statistical analyses were performed in PAST 3.22 [38]. Unfortunately, due to time constraints, inter-observer error was not tested.

## Results

The results of the repeatability equation demonstrated that aside from the perimeter of the nasal aperture, which had a repeatability of 91%, all other measurements had a repeatability above 95%, providing an average repeatability of 97%.

The results of all the PERMANOVA tests and pairwise *post-hoc* analyses are summarised in Tables 5 and 6, respectively. Overall, there is a significant difference (*P* < 0.05) between groups, regardless of the category or combination of 3D measurements considered in the test (Table 5). The pairwise *post-hoc* analyses (Table 6) indicate that the individuals of European descent are statistically different (*P* < 0.05) from those of African and Canadian Inuit descent for all tests. However, the African-derived group is not statistically different (*P* > 0.05) from the Canadian Inuit for all tests except proportion ratios.

**Table 5.** PERMANOVA results per measurement category.

Comparison	*F*-value	*P*-value
*All measurements*	8.024	0.0003*
*Circumference/perimeter*	4.799	0.0037*
*Surface area*	8.028	0.0001*
*Proportion ratios*	14.36	0.0001*

* Significant at α = 0.05.

**Table 6.** Pairwise PERMANOVA post-hoc results per measurement category.

Pairwise post-hoc analyses	*P*-value
*All measurements*	
African × European	0.0009*
African × Canadian Inuit	1
European × Canadian Inuit	0.0015*
*Circumference/perimeter*	
African × European	0.0075*
African × Canadian Inuit	0.8793
European × Canadian Inuit	0.0492*
*Surface area*	
African × European	0.0012*
African × Canadian Inuit	1
European × Canadian Inuit	0.0009*
*Proportion ratios*	
African × European	0.0003*
African × Canadian Inuit	0.0033*
European × Canadian Inuit	0.0003*

* Significant at *α* = 0.05 (following Bonferroni correction).

The results of all the discriminant function analyses are summarised in Tables 7–9. All discriminant analyses reveal two discriminant functions, regardless of the category or combination of 3D measurements considered in the test (Table 7). The discriminant analysis providing the highest classification rate is the one containing all 3D measurements, rather than a single measurement category (Table 8). This function has a classification rate of 87.36%, although it drops to 80.46% when a jackknifed confusion matrix is used. All other measurement categories, when evaluated individually, provide classification rates below 75%. Individuals of African descent could not consistently be distinguished from Canadian Inuit (Table 9), while those of European descent overlap with African or Canadian Inuit, depending on the measurement category.

**Table 7.** Discriminant function analysis (DFA) results per measurement category.

Function	Eigenvalue	% of variance
*All measurements*		
**1**	2.4022	82.32
**2**	0.51603	17.68
*Circumference*		
**1**	0.48123	98.89
**2**	0.0053896	1.108
*Surface area*		
**1**	0.3716	96.89
**2**	0.011924	3.109
*Proportion ratios*		
**1**	0.99098	71.08
**2**	4 031	28.92

**Table 8.** Discriminant function analysis (DFA) classification rates per measurement category.

Comparison	Classification rate (%)	Jackknifed (%)
*All measurements*	87.36	80.46
*Circumference/perimeter*	63.22	60.92
*Surface area*	56.32	52.87
*Proportion ratios*	73.56	64.37

**Table 9.** Discriminant function analysis (DFA) confusion matrices per measurement category.

Actual population affinity		Classified population affinity (jackknifed)*		
Group	*n*	African	European	Canadian Inuit
*All measurements*				
African	13	11 (9)	0 (2)	2 (2)
European	50	2 (3)	45 (43)	3 (4)
Canadian Inuit	24	3 (4)	1 (2)	20 (18)
**Total**	**87**	**16 (16)**	**46 (47)**	**25 (24)**
*Circumference/perimeter*				
African	13	8 (8)	1 (1)	4 (4)
European	50	4 (4)	34 (34)	14 (12)
Canadian Inuit	24	8 (9)	3 (4)	13 (11)
**Total**	**87**	**20 (21)**	**38 (39)**	**29 (27)**
*Surface area*				
African	13	9 (8)	2 (2)	2 (3)
European	50	8 (8)	33 (32)	9 (10)
Canadian Inuit	24	11 (11)	6 (7)	7 (6)
**Total**	**87**	**28 (27)**	**41 (41)**	**18 (19)**
*Proportion ratios*				
African	13	10 (9)	1 (2)	2 (2)
European	50	6 (7)	37 (33)	7 (10)
Canadian Inuit	24	4 (6)	3 (4)	17 (14)
**Total**	**87**	**20 (22)**	**41 (39)**	**26 (26)**

* Raw values are shown first, without parentheses; jackknifed values are shown second, in parentheses.

The discriminant function plots (Figure 10) corroborate these findings, demonstrating some inter-group differences, but mostly overlap between groups for single measurement type categories. The plot containing all 3D measurements (Figure 10A) is the only plot indicating a clear distinction of the European (yellow — left half), African (orange — upper right) and Canadian Inuit (blue — lower right) populations, as more variables are included in this plot. The circumference/perimeter and surface area plots (Figure 10B and C) lack a clear distinction of groups, while in the proportion ratios plot, some distinction of groups is visible (Figure 10D).

## Discussion

Despite representing a small percentage of the total Canadian female population, the RCMP has demonstrated that Canadian Indigenous women and girls are at a higher risk of being victims of violence than other women in Canada and are over-represented as victims of homicide [2, 3]. Some risk factors that have been associated with violent victimisation of Canadian Indigenous women and girls include drug use, binge drinking, fair or poor mental health, some form of disability and a history of homelessness [39]. However, even when considering such factors, the high victimisation rates seen among Canadian Indigenous women and girls cannot be fully explained, thus highlighting the need for more research on this matter [39].

The RCMP’s study [2, 3] was a step forward in the right direction towards finally addressing the issue of MMIWG in Canada; however, a major issue with their findings is that the data were derived from various databases of police reports where the identification of Indigenous biogeographic origin was inconsistent. As per the RCMP’s 2014 operational overview [2], the use of the term “Aboriginal” as a means to determine identity varied between different data sources, police jurisdictions, agencies, officers, inspectors, and even among the family and friends of the victims. Population affinity was therefore not determined from a biological perspective, and it is possible that a significantly higher number of MMIWG remain unidentified in numerous medical examiners and coroners’ offices. A forensic anthropological analysis including the assessment of population affinity as part of the biological profile could therefore address unsolved cases containing human skeletal remains, with the hopes of increasing the identification of MMIWG.

The variables presented here demonstrate the ease and value of quantifying morphological variation that is both accurate (80.46% on jackknifed samples) and precise (>91% repeatability) in a manner that is easily standardised. To be considered valid for court purposes, a method must be assessed for accuracy and precision. In forensic anthropology, a ≥80% threshold is generally accepted as a standard for accuracy, while a ≤10% threshold is generally accepted for precision [22], both of which are met by this study’s results. Other techniques, such as optimised summed scored attributes (OSSA) [18], have also reported acceptable classification accuracies (>85% for OSSA), and could be considered for the morphological assessment of population affinity in cases. That being said, some issues have been found with OSSA, such as experience still being required to accurately attribute the character state scores and some traits performing lower than reported due to difficulties in ranking them consistently [40].

Although the current research achieved acceptable accuracy and precision scores, it is important to note that these standard protocols may have some element of subjectivity. Using the approach described here, tracing orbit, nasal and palate shapes can be influenced by the observer’s selective placement of the vertices on the 3D model to create the outline. To some degree, this issue can be mitigated by standardising the real-world and virtual workspaces, as described in the methodology. It is expected that minor fluctuations in placement of the vertices will not significantly impact the results, but a series of inter-observer error tests are required to confirm. Whether other observers, both familiar and unfamiliar with the 3DS Max software, can reproduce results will be key in establishing the method’s reliability in the field and in the courtroom [41]. If the technique is shown to have a high repeatability between observers, it will contribute to making population affinity assessments less dependent on an anthropologist’s experience with human variation, as has been heavily criticised in the past [6]. Hence, this technique can help assessments become more accessible to young forensic anthropologists and support their analyses as they build their experience with human variation.

While the method described here was initiated with photogrammetry, any 3D imaging system can be used to acquire the image. One benefit of photogrammetry is that it can be completed using a standard DSLR, and even high-quality phone cameras. In our current digital era, the use of digital technology, particularly 3D imaging, has numerous benefits compared to traditional morphological assessment methods. Three-dimensional imaging provides a high-definition digital copy of the skeletal remains, which offers a permanent archival record [24, 42]. In forensic contexts where skeletal remains are limited in quantity, fragile and/or difficult to access, digital archiving is a highly desirable feature, particularly since it is non-invasive and non-destructive, and therefore maintains the integrity of the bones [42]. Relevant to this research, 3D models also allow for the accurate quantification of angles, surface areas and volumes through the extraction of coordinate data, which are most useful for the computation of precise statistical shape analyses [24]. These values cannot be readily obtained using traditional methods. Developing metric analyses specific to 3D models provides a valuable suite of new tools for forensic anthropologists, particularly as 3D imaging becomes a standard part of case documentation. Similarly, in cases where defence lawyers seek an independent forensic anthropological evaluation of the evidence, which often occurs well after the remains have been returned to the family, analyses can be completed from images or digital models [43].

One of the main limitations of this research was the sample size, which totaled 87 individuals. The European-derived group was well-represented (*n* = 50), but the Canadian Inuit (*n* = 24) and African-derived (*n* = 13) groups were significantly smaller. Their small sample sizes limited the variation captured and may have contributed to some of the overlap between groups. As this research was focused on MMIWG in Canada, the most significant population to include in the sample was Canadian Indigenous women. However, recent developments in Canadian Indigenous relations and the establishment of new policies regarding Indigenous rights at the time of the study made it particularly difficult for the researcher to gain access to Indigenous remains, thus leading to a significantly smaller and less diverse Canadian Indigenous sample size. At the international level, the United Nations Declaration on the Rights of Indigenous Peoples (UNDRIP), published in March 2008, presented a set of standard rights, through 46 articles guided by the Charter of the United Nations, which all countries home to Indigenous communities are required to abide by [44]. Relevant to this research, UNDRIP calls for the repatriation of human remains, as stated in Article 12:

“1. Indigenous peoples have the right to manifest, practise, develop, and teach their spiritual and religious traditions, customs, and ceremonies; the right to maintain, protect and have access in privacy to their religious and cultural sites; the right to the use and control of their ceremonial objects; and the right to the repatriation of their human remains.

2. States shall seek to enable the access and/or repatriation of ceremonial objects and human remains in their possession through fair, transparent and effective mechanisms developed in conjunction with Indigenous peoples concerned.” [44,p.6]

In Canada specifically, the rights of Indigenous communities have also been addressed by the Truth and Reconciliation Commission (TRC), which was established to redress the legacy of residential schools and advance the process of Canadian reconciliation [45]. The outcome of the TRC was a significant number of calls to action to the federal government in 2015, covering areas such as child welfare, education, language and culture, health, and justice [45]. Relevant to this research, the TRC called for a public inquiry into the causes of the disproportionate victimisation of Aboriginal women and girls, and particularly, an investigation into missing and murdered Aboriginal women and girls, as part of its requests regarding justice [45]. However, as part of reconciliation measures, the TRC also requested that the federal government provide funding to the Canadian Museums Association to undertake, in collaboration with Indigenous communities, a national review of museum policies and best practices to determine the level of compliance with UNDRIP and to then make recommendations on its findings [45].

This demand directly impacted the current study, as the CMH’s policies sporadically changed to abide by the TRC request as the project advanced, which led to additional delays in data collection and analysis. As responses from the various Indigenous communities were received, the final sample size for the Canadian Indigenous was reduced to only five women from Alberta and Manitoba. Given the likelihood of unreliable statistical results that would be brought about by the very low sample size, it was decided to exclude those Canadian Indigenous women from the analyses and the study instead became focussed solely on the Canadian Inuit individuals available. Although Canadian Inuit are said to be morphologically different from other Canadian Indigenous groups [46], the high number of MMIWG who may identify as Inuit is equally important to address as part of the issue happening in Canada. Unfortunately, it is most likely that the current findings are only applicable to Canadian Inuit women and further research would be needed to capture a wider range of variation for the Canadian Indigenous population.

Increasing sample size, as well as the number of groups considered, would provide more robust statistical results, and capture a wider range of human variation [47]. Incorporating Asian samples in order to differentiate more clearly between North American Indigenous and the broader Asian parent population would be particularly useful [48], as would incorporating under-represented Indigenous groups, such as Australian Aboriginals [49], and more recently admixed populations, such as Hispanics [50]. In addition, this study focused on females only. Given the relationship between sex and population affinity, particularly with respect to size [13], it is important to expand this research to include males, and to examine the success of this approach when sex is not known.

In summary, the results presented here show that the technique is satisfactory in correctly evaluating population affinity with an accuracy of 87.36% (jackknifed: 80.46%) and an average repeatability of 97%, both of which are acceptable in terms of method validity for court purposes. However, the small Canadian Indigenous sample size impacted the applicability of the results and further research will be required before the technique can be used to aid in the identification of MMIWG in Canada. Sadly, the recent discoveries at former residential schools’ burial grounds across Canada have reminded the entire nation that much work remains to be done before reconciliation with the Indigenous communities can be achieved. One can only hope that the issue of MMIWG will have its place in future political and social discussions regarding elements that need to be addressed in order to provide a better future for Indigenous communities in Canada.
